# Determination of moderate walking intensity using step rate and VO_2_ reserve in healthy men

**DOI:** 10.1186/s12889-024-17843-0

**Published:** 2024-02-07

**Authors:** Woo Ram Bae, Yongsuk Seo, Somi Yun, Dae Taek Lee

**Affiliations:** Exercise Physiology Laboratory, Kookmin University, Seoul, Republic of Korea

**Keywords:** Walking, Moderate intensity, Physical activity

## Abstract

**Objective:**

This study investigated step rates (SR) during overground walking to estimate the relative aerobic capacity that corresponds to a moderate intensity.

**Methods:**

The present study utilized a repeated measure, within-subjects design incorporating a counterbalanced order. A total of twenty-three healthy men walked on a 119-meter oval track with artificial turf at self-selected pace (FP), 100, 120, and 140 steps/min for 6 min each while oxygen uptake (VO_2_), speed (in km/h), distance (in m), and steps (in steps/min) were measured.

**Results:**

During FP, participants walked an average cadence of 117 ± 9.3 steps/minclose to 120 steps/min, which corresponds to 4.7 metabolic equivalents (METs). The estimated VO_2_ reserve was 30.5% of VO_2_ reserve at the FP and was close to the 120 steps/min of 33.3%. At the 100 and 140 steps/min, the VO_2_ reserve were 24.1% and 45.2%, respectively. The regression analysis revealed that an SR of 88.2 elicited 3METs and 17.1% of VO_2_reserve. Additionally, an SR of 129 elicited 5.9METs and 40% of VO_2_ reserve.

**Conclusions:**

This study demonstrated that a moderate walking intensity for young, healthy men corresponded to 128.9 steps per minute. A range of 120 ~ 140 steps/min for walking could be recommended as a general guideline for moderate-intensity exercise. However, concerning providing public guidelines, caution should be taken regarding determining the moderate walking intensity due to the individual’s fitness level.

## Introduction

Regular physical activity has been reported to reduce the risks of cardiovascular disease, diabetes mellitus, certain types of cancer, and obesity [1–4]. Furthermore, the American College of Sports Medicine (ACSM) and the American Heart Association jointly recommend engaging in moderate-intensity aerobic activity for more than 30 min a day on more than 5 days per week, or vigorous-intensity activity for more than 20 min a day on more than 3 days per week, to promote and maintain health and fitness [5]. Physical activity guidelines for the prescription of aerobic exercise involve the deliberate adjustment of training variables, encompassing training frequency, intensity, duration, and modality, in alignment with the individual’s age, baseline fitness level, and pertinent clinical considerations [6, 7]. ACSM defined the three main components of energy expended during exercise: frequency, duration, and intensity [8]. While the frequency and duration of physical activity are assumed to be valid in both epidemiological and clinical contexts, the process of determining and quantifying the intensity of physical activities remains somewhat ambiguous [9–11]. As there are dose-response relationships between physical activity and health [5, 7], achieving optimal health benefits might necessitate enhanced precision in estimating and engaging in physical activity.

Many tools and methods are available for estimating the level of exercise intensity using talk ability, heart rate, perceived effort, oxygen uptake (VO_2_), and motion sensor [12]. One of the frequently utilized methods is the ACSM guideline, which involves the utilization of heart rate reserve (HRR) and oxygen uptake reserve [8]. ACSM guidelines provide a specific range of HRR and %VO_2_reserve While this approach proves effective in estimating the desired exercise intensity, these methodologies necessitate intricate protocols, considerable time investment, and skilled personnel. An alternative method of gauging exercise intensity involves evaluating speech capacity to exertion, commonly referred to as the Talk Test [13]. This method is believed to offer an estimation of the ventilatory threshold during exercise, characterized by its simplicity, practicality, and validity [13–15]. However, a previous study reported that the Talk Test revealed weak inter-tester reliability with cardiac patients [16].

Scruggs, et al. proposed a step rate (SR) to estimate the exercise intensity during walking. The SR is defined as step frequency/min and is relatively simple, easy to follow, and accurate to count [17–19]. Several previous studies reported that SR provides useful information for predicting moderate walking intensity. Previous studies determined the exercise intensity that 100 steps/min matches a minimum level of moderate walking intensity, reaching 3 metabolic equivalents (METs) [18–22]. However, their experimental trials were conducted in well-controlled laboratory setting with a treadmill. It would be more reasonable to determine the walking intensity during ground walking for practical application [23, 24].

Therefore, the purposes of this study were (1) to examine the walking intensity during free walking for young healthy men and (2) to identify the minimum SR required to achieve a moderate walking intensity, as determined by relative VO_2_. It was hypothesized that walking at an approximate pace of 100 steps/min corresponds to a moderate level of physical activity intensity.

## Methods

### Participants

A total of 23 healthy young men (mean ± standard deviation; age 25.3 ± 1.8 years; height 175 ± 5.5 cm, weight 75.7 ± 14.2 kg; BMI 21.6 ± 3.6 kg/m^2^ and VO_2_peak 41.0 ± 6.3 ml/kg/min) volunteered to participate in the current study. The sample size was determined via Gpower software (ver. 3.1.9.2) with an assumed power of 0.8 and an effect size (Cohen’s d) of 0.7. It was concluded that recruiting 20 participants would be necessary to attain statistical significance in the comparison of SR. 

All participants were screened through a health history questionnaire before participation and were excluded if they reported the presence of cardiovascular and musculoskeletal disorders as well as recent injuries within the past three months. Each participant signed a written and verbal informed consent and completed the Physical Activity Readiness Questionnaire before participation. All subjects were free of any cardiac, metabolic, or respiratory disease, and any musculoskeletal issues prohibiting exercise. The Institutional Review Board at Kookmin University Research Ethics Committee (KMU-201,710-HR-163) approved this study.

### Experimental design

The current study employed a repeated measure, within-subjects design with counterbalanced order. 

Participants underwent two experimental occasions which consisted of a prescreening session and subsequent four experimental sessions, each separated by at least 7 days to ensure full recovery. During the prescreening session, their physical characteristics and fitness were assessed. At each experimental session, participants walked at one of four different step rates of free paced (FP), 100 (100SR), 120 (120SR), and 140 steps/min (140SR).

The metronome was used to establish the step rate and three minutes of rest was given between the step rates.

### Experimental procedure

Before participation, participants were screened with a medical questionnaire and familiarized with the study protocol. During the prescreening session, body weight, height, and body composition were assessed using a bioimpedance method (BSM330, Inbody, Seoul, Korea). The participants rested in a seated position for 15 min for resting heart rate (HR) and VO_2_ were taken. Resting HR and VO_2_ were considered the lowest values during the resting. After resting, participants performed a VO_2_peak test with modified Bruce Protocol on a treadmill (T170 DE, Cosmed, Fridolfing, Germany) using an automatized spirometer (Jaeger Oxycon Mobile, Würzburg, Germany).

To determine VO_2_peak, the participants performed incremental running until volitional fatigue. The volitional fatigue was considered when three of the five criteria were satisfied; (1) no increase in HR as the workload increased, (2) when the respiratory exchange ratio exceeded 1.15, (3) when the rating of perceived exertion (RPE) was > 17 on a scale of 6–20, (4) no increase in VO_2_ as the workload increased, and (5) when participant called give-up [25, 26].

On the day of the four experimental session, participants reported to the Exercise Physiology Laboratory at Kookmin University. The participants were equipped with an HR monitor (M400, Polar, Oulu, Finland), two pedometers (HJ-720ITC, Omron, Tokyo, Japan) on the right and left waist, and a mask connected to a gas analyzer. The gas analyzer was calibrated each time before the first walk. During the first walking, the participants were free to choose a speed and cadence at which they felt was a moderate intensity for 6 min.

HR and VO_2_ were monitored continuously and an average of the final 2 min was considered the steady state HR and VO_2_. A steady state HR was also considered when the variation in HR was < 5 bpm during the period. Metabolic equivalents (METs) was calculated during walking and individual resting VO_2_ was considered 1MET [27]. The moderate intensity in METs was defined as 3.0 ~ 5.9METs and relative exercise intensity in %VO_2_reserve was defined as 40 ~ 59 [8].

The total number of steps was determined by the average of the two pedometers, and the total walking distance (WD) was measured. SR in steps/min, step length (SL) in meters, and walking speed (WS) in km/h were calculated for 6 min. Once the first trial was completed, the participants rested for 3 min and then started another trial. The procedure and measurements were identical to the first trial, except for RPE. The subjective measurement of exertion was recorded using the Borg perceived Exertion Scale of 6–20 [25].

### Statistical analysis

Statistical analyses were performed using Statistical Package for the Social Sciences 22.0 (IBM-SPSS, Somers, NY, USA). Using SPSS 22.0, a one-way analysis of variance was performed to compare the walking intensity by SR. Scheffe’s post-hoc test was employed to determine if there were significant differences at each SR. A predictive equation was developed by linear regression to identify a cut-off point for moderate exercise intensity across all step rates. For all analyses, significance was set at an alpha level of ≤ 0.05 and all data are expressed as means ± standard deviations.

## Results

### Walking characteristics

**Fig. 1:**
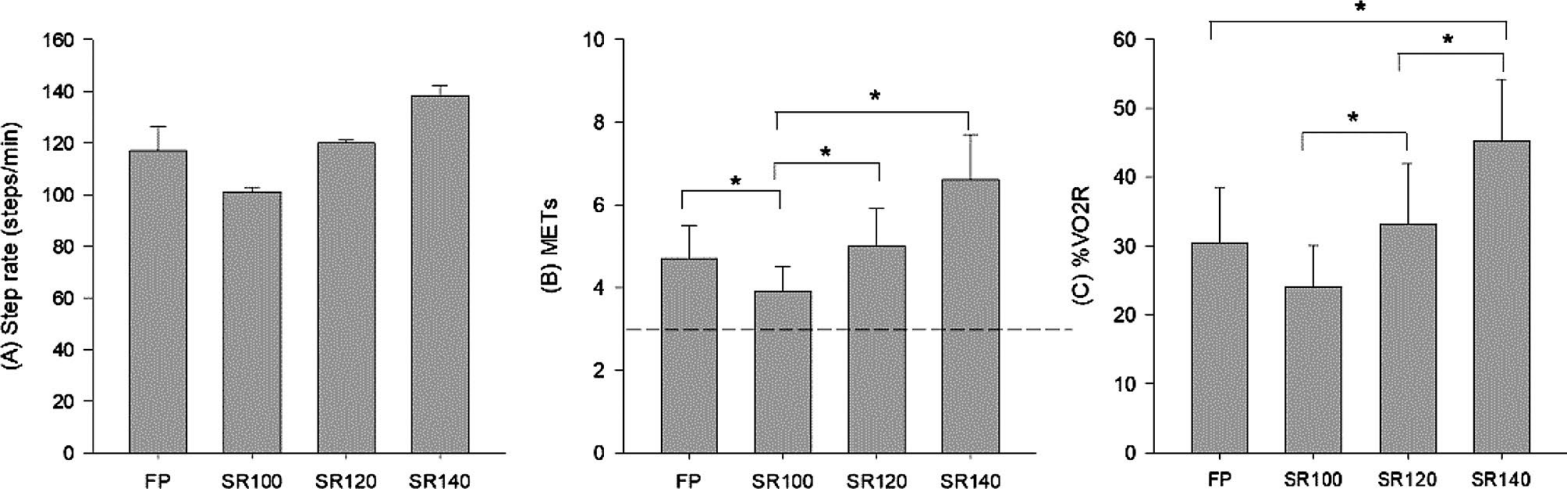
**(A)** step rates, **(B)** metabolic equivalents (METs), and **(C)** percent VO_2_ reserve during walking at different step rates. Values are mean ± standard deviation. FP: free-paced walking, SR100: step rate at 100 steps/min, SR120: step rate at 120 steps/min, SR140: step rate at 140 steps/min. The dotted horizontal line in Figure **(B)** indicates defined moderate intensity (≥ 3METs). * Denote a significant difference between step rates (*p* ≤ 0.05)

The SR, METs, and %VO_2_reserve are shown in Fig. 1. The average SR were 117.0 ± 9.3, 100.9 ± 98.4, 119.7 ± 1.1, and 138.0 ± 3.9 steps/min in the FP, SR100, SR120, and SR140, respectively (Fig. 1A). The measured METs were significantly lower in SR100 (3.9 ± 0.6) compared to FP (4.7 ± 0.8), SR120 (5.0 ± 0.9), and SR140 (6.6 ± 1.1) (all *p* ≤ 0.05) (Fig. 1B). At an average METs, SR100 and SR120 were categorized as moderate intensity (3 ~ < 6METs), while SR140 was classified as high intensity (≥ 6METs). The %VO_2_reserve was significantly lower in SR100 (24.1 ± 6.0) compared to FP (30.5 ± 8.1), SR120 (33.3 ± 8.6), and SR140 (45.2 ± 9.0) (all *p* ≤ 0.05) (Fig. 1C).

**Fig. 2:**
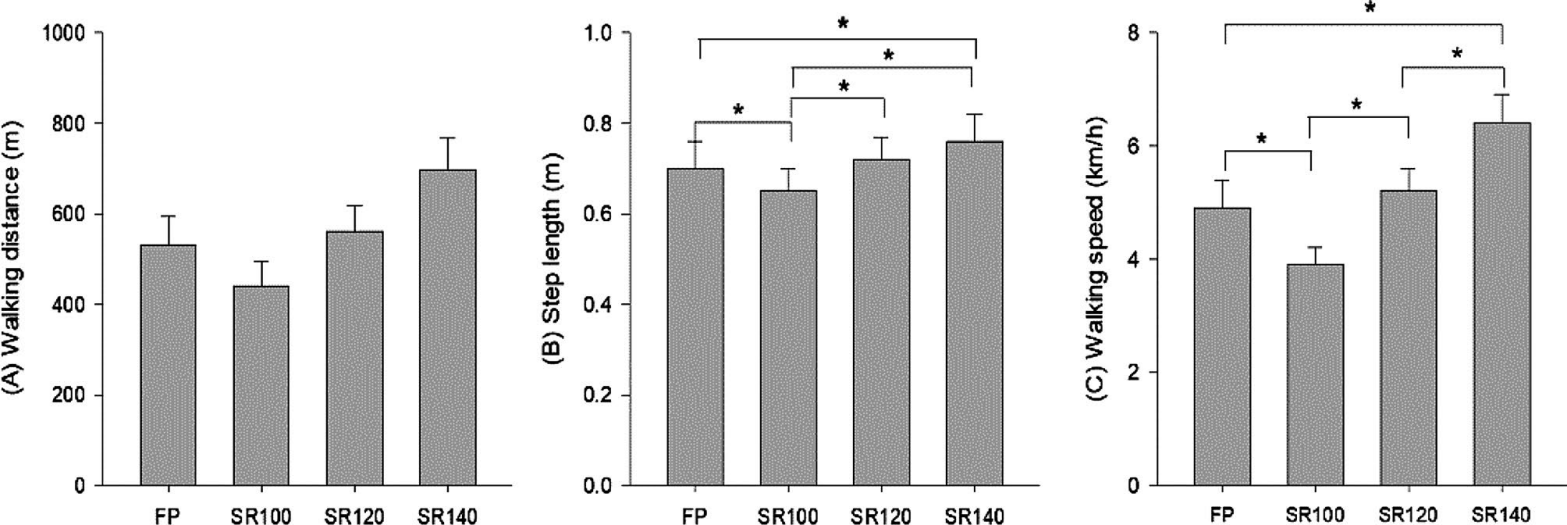
**(A)** walking distance, **(B)** step length, and **(C)** walking speed at different step rates. Values are mean ± standard deviation. FP: free-paced walking, SR100: step rate at 100 steps/min, SR120: step rate at 120 steps/min, SR140: step rate at 140 steps/min. * Denote a significant difference between step rates (*p* ≤ 0.05)

The WD was significantly lower in SR100 (441 ± 55 m) compared to FP (530 ± 63 m), 120SR (561 ± 58 m), and SR140 (696 ± 70 m) (all *p* ≤ 0.001) (Fig. 2A).

The average SL was 0.70 ± 0.06, 0.65 ± 0.05, 0.72 ± 0.05, and 0.76 ± 0.06 m in FP, SR100, SR120, and SR140, respectively. The SL in SR100 was significantly lower compared to FP, SR120, and SR140, respectively (all *p* ≤ 0.05) (Fig. 2B). The WS in SR100 (3.9 ± 0.3 km/h) was significantly lower compared to FP (4.9 ± 0.5 km/h), SR120 (5.2 ± 0.4 km/h), and SR140 (6.4 ± 0.5 km/h) (all *p* ≤ 0.001) (Fig. 2C). RPE was significantly higher in SR120 (9.4 ± 2.0) and SR140 (12.3 ± 1.9) compared with SR100 (7.4 ± 1.5) (all *p* ≤ 0.05), indicating that SR140 was classified as moderate intensity.

### Linear regression analyses

The linear regression analysis was employed to establish a regression equation that can be utilized to determine the moderate intensity cut point for step rate. Equations 1 and 2 presents the linear regression equation between SR and exercise intensity (METs and %VO_2_reserve).

A simple linear regression analysis for SR indicated a significant correlation with METs (*p* ≤ 0.001) and %VO_2_reserve (*p* ≤ 0.001). The results of the linear regression indicated that 88.2 steps/min and 140 steps/min elicited 3METs and 45.2% of VO_2_reserve, respectively.

1 $$METs\, = \, - \,3.188591\, + \,\left( {0.0701456\, \times \,SR} \right)$$

2 $$\% V{O_2}R\, = \, - 32.33999\, + \,\left( {0.5615589\, \times \,SR} \right)$$

## Discussion

This current study determined SR representing a moderate exercise intensity during walking on a flat surface in the healthy population. It was hypothesized that SR at 100 steps/min would correspond to moderate exercise intensity. The main findings of the current study were (1) the previously suggested SR of 100 steps/min was found to be lower than the FP walking in this study, (2) linear regression revealed a slightly lower cut-off point of 88.2 steps/min for moderate intensity (3METs), while the minimum threshold for moderate intensity based on %VO_2_reserve was determined to be 128.9 steps/min.

Previous research has shown that SR serves as a readily accessible metric for assessing ambulatory behavior. To elucidate further, when the duration of walking and the total number of steps taken are established, it becomes feasible to compute the intensity of physical activity [11, 21, 28]. Furthermore, previous studies indicated that walking at 100 steps/min corresponds to moderate intensity [19, 29]. However, our results indicated a SR cut point was 88.2 steps/min for moderate intensity. This SR is somewhat lower than previous findings [19, 29]. One plausible explanation for the observed discrepancy could be linked to the comparatively lower direct measurement of resting metabolic rate in the present study, which was recorded at 3.1 ml/kg/min instead of the expected 3.5 ml/kg/min. This lower resting metabolic rate measurement may have led to an underestimation of exercise intensity. Additionally, variations in physical characteristics such as height, weight, and fitness levels among participants may have contributed to this difference. It is important to note, SR100 was equivalent to 3.9METs which falls within the range of moderate intensity (3 ~ 6METs). However, other parameters such as WS, RPE, and %VO_2_reserve indicated lower intensity. Indeed, the WS of 100SR elicited 3.9 km/h, which was lower than the recommended moderate intensity [30].

The average SR of the participants during FP in this study was 117.1 ± 9.3 steps/min. This is similar to the results of previous findings [31], where 70 young adult participants, consisting of 34 males and 36 females, walked at their self-selected normal walking pace for 5 min, resulting in cadences of 114.2 ± 4.3 steps/min for males and 116.3 ± 4.7 steps/min for females. These findings are also in line with a previous study by Kim and Kim [32], who reported an average cadence of 112.67 ± 5.2 steps/min when 15 young adult males walked at their self-selected moderate walking intensity [32].

The recommended SR of 100 steps/min for moderate-intensity walking appears to be lower than the SR observed during free walking in this study. These findings emphasize the need for thoughtful consideration when determining walking intensity based on SR. For example, suggesting a SR of 100 steps/minute for someone accustomed to walking at 115 steps/minute would recommend a lower intensity than their usual walking pace.

As seen in the results of this study, the SR during FP and at SR120 showed no statistically significant difference (117.1 ± 9.3 vs. 119.7 ± 1.1 steps/min), and there was no significant difference in stride length either (0.70 ± 0.06 vs. 0.72 ± 0.05 m). When SR and SL are equal, the distance is covered in the same amount of time, resulting in similar walking speeds. There was no significant difference in WS between the two conditions in this study (4.9 ± 0.5 vs. 5.2 ± 0.4 km/h).

Relative intensity is valuable for establishing appropriate activity levels for individuals as they evaluate activity intensity with an individual’s maximum capacity. Oxygen uptake is considered the gold standard for accurately measuring energy metabolism during physical activities [33]. When applying a minimum VO_2_reserve threshold of 40% as indicative of moderate exercise intensity, the study results reveal that the following conditions did not meet the criteria for moderate intensity: FP (30.5 ± 8.1%), SR100 (24.1 ± 6.0%), and SR120 (33.3 ± 8.6%). However, when walking at an SR140, the criterion for moderate intensity was met (45.2 ± 9.0%). ACSM [8] suggested that a VO_2_reserve of 40% is the lowest bound of moderate intensity [8]. Furthermore, findings by Serrano et al. indicate that a mean walking cadence of 115 ± 10 steps/min is necessary to attain a VO_2_reserve of 40% [34]. This observation suggests that external measures of exercise intensity, such as accelerometry, may underestimate the requisite walking cadence to reach moderate-to-vigorous physical activity levels [35].

### Limitations

This study has several limitations that warrant consideration for generalization and interpretation. Firstly, the use of a limited sample consisting of young, healthy, physically active men may restrict the applicability of the findings to broader populations. To enhance external validity, future research should encompass larger and more diverse groups, including older adults and women.

Furthermore, it’s crucial to note that five participants experienced less than 40% of their maximal aerobic capacity even at a cadence of 140 steps/min during walking. This observation implies that while fast walking could be advantageous for the elderly, sedentary individuals, and those with obesity, it may not pose a sufficient challenge for young, healthy adults [36]. Consequently, jogging or running might be more suitable for achieving moderate-intensity exercise in this population.

Additionally, the study underscores the importance of considering body weight and height when adopting a specific step rate. A prior study by Marchall et al. estimated SRs to elicit 3METs, revealing differences for normal, overweight, and obese participants with values of 127, 94, and 103 steps/min, respectively [29]. Given that overweight and obese participants often exhibit lower fitness levels [37, 38], achieving a fast cadence among this group may present challenges. In our study, two participants with a BMI over 25 kg/m² struggled to maintain a pace of 140 steps per minute and could only reach the 130 steps/min threshold. Consequently, there is a need for investigations in larger and more diverse populations to comprehensively explore exercise intensity and its implications, ensuring a more inclusive understanding across a broader range of population.

Strengths of this study are worth noting. First, this study design allowed for the comparison of four different step rates. Second, the present study was measured in field settings, which provide practical implications to identify the step rate cut points for exercise prescription.

In conclusion, this study reveals that for healthy young men, a moderate walking intensity corresponds to a cadence of 128.9 steps/min when employing the oxygen uptake reserve calculation. The findings also demonstrated that establishing a single universal criterion for practical public guidelines is not feasible, given the variations in fitness levels among individuals.

## Data Availability

The datasets used and/or analysed during the current study available from the corresponding author on reasonable request.
